# Research on identity authentication and data security of photovoltaic power generation management personnel based on blockchain technology

**DOI:** 10.1371/journal.pone.0323340

**Published:** 2025-05-29

**Authors:** Fan Xu, Dengping Zhang

**Affiliations:** 1 State Power Investment Ronghe Investment Co., Ltd., Qinghai Huanghe Hydropower Development Co., Ltd., Xining, Qinghai, China; 2 Sinohydro Engineering BUREAU 4 Co., Ltd., Power Construction Corporation of China, Xining, Qinghai, China; Dayananda Sagar College of Engineering, INDIA

## Abstract

As high-frequency technological developments happen and the variety of Microgrids (MG) grows, microgrids are going to be able to secure their own usage while competing in the electricity sector. To optimize each individual’s earnings, the most efficient bidding approach for the transaction process is determined using a novel customized hunter prey optimization (CHPO), it has been established with blockchain technology and shown as a MG transactional framework in this research. To address the issue of energy underuse in a MG gaming competition, research have developed the CHPO, an innovative technique in the most effective bidding approach for the transaction process. Two market participants such as Major Users (MU) and Users of Microgrid (UoM) were explored for the performance in energy transactions. The market participants performance was creating various methods for buying and selling power at various locations in the MG framework to enhance the participant’s desires and power developments in the market. In addition, the main market participants are employed as study subjects to develop a distributed energy resources (DERs) integration system that addresses the issue of real-time power pricing. The design comparison is analyzed with an authentication paradigm to demonstrate its applicability to microwave, optical, and radio frequency (RF) technologies. In addition, the main market participants are employed as research subjects to develop a DERs integration system that addresses the issue of real-time power pricing. The design comparison is analyzed with an authentication paradigm to demonstrate its applicability to microwave, optical, and RF technologies. The CHPO method demonstrated high efficiency with a mean convergence time of 54 iterations and an interval of 240, which makes it scalable. It ensures low latency at 45ms, and the unit operation metrics (UoM) range from 2,402,077–2,935,889, which is quite robust. The CHPO method also performed better in RMSE at 4.75, MAPE at 10.12, MAE at 2.31.

## Introduction

**Fig 1:**
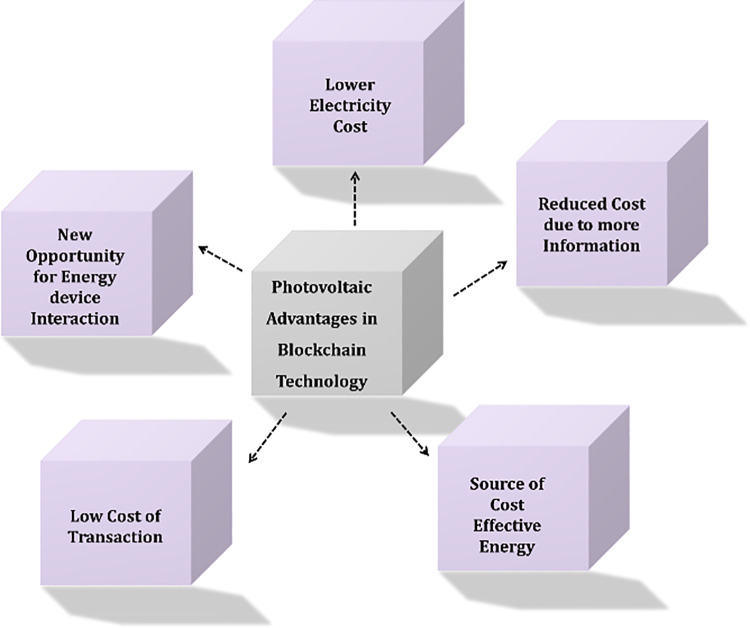
Advantages of Blockchain Technology inPhotovoltaic Power.

Photovoltaic systems perform most effectively in remote areas without connection to the national network. Utilizing photovoltaic systems to power farms has several major benefits, including the small number of components and limited space requirements of network-connected solar systems, designing can be made easier of annual power usage is known as photovoltaic [1]. Blockchain technology is very useful for improving the efficacy of photovoltaic systems by offering secure, decentralized platforms for managing energy transactions and maximizing the integration of RE resources. Its tamper-proof nature provides openness in energy distribution and allows for real-time cost modifications. Energy distribution and management in the energy sector has evolved from centralized to hierarchical models. The decentralized energy industry has seen a number of problems, such as the necessity to store customer data and maintain data quality, fairness, and reliability during the transaction phase [2]. Large-scale power systems have been made in several areas, and the electrification process has improved rapidly. The system with several dispersed power sources and associated services that maintain a particular topological structure is called the microgrid (MG) [3]. MG that are composed of several components including DERs with coordinated control and interconnected applications [4] enhances the reliability, dependability, and performance of such modern photovoltaic power systems. Blockchain’s capacity to support safe energy transactions makes it ideal for MG systems, especially in terms of increasing the efficiency of DERs and tackling the issues of real-time power cost control. Utilizing information and communication technology (ICT), smart energy management (SEM) is required to assess and coordinate the requirements and capacities of all sources and resources, energy transforms, infrastructure operators, end users, and stakeholders in the energy market [5]. The operation, treatment, and development of the conventional electrical grid are severely limited [6]. By facilitating peer-to-peer energy transfers and enhancing system resilience through decentralized architecture, integrating blockchain technology into solar systems offers a way around these restrictions. Developments in energy consumption begin to rise approximately in combination with economic growth as economies evolve. It causes a strong and positive relationship between energy use and economic growth [7]. A system design that incorporates several well-known technologies, like distributed environments, smart contracts, cryptography, acceptance methods, peer-to-peer networking, and decentralized architecture is facilitated by the blockchain [8]. Fossil fuels, solar power and RE are the three kinds of energy sources that provide global energy needs. Nuclear energy and fossil fuels can be reliable and cost-effective [9]. Traditional power networks are capable of distributing constant amounts of electricity in instances as it is uncertain the amount of usage in a specific region [10]. Photovoltaic systems convert sunlight into electricity, providing a renewable and eco-friendly energy source. However, their intermittent nature can affect grid stability. Battery Energy Storage Systems (BESS) store excess energy and release it when needed, ensuring continuous power supply. Composed, Photovoltaic and BESS enhance energy reliability, efficiency, and Renewable Energy (RE) integration in MG. The objective of the research is the blockchain-based transaction design employing an innovative customized hunter-prey optimization (CHPO) to improve S MG reliability and competitiveness in the field of electric power. It enables reliable converging and real-time power cost while managing power inactivity. The advantages of blockchain technology in photovoltaic power are illustrated in Fig 1.

### Contributions of the research

- A distributed resource integration system that examines the issues of the real-time power market is being developed and the major participants in the market are being used as research participants.
- To optimize their individual desires and impact on market developments, users of Microgrid (UoM) and Major Users (MU) are developing innovative methods to purchase and sell power at numerous locations within the MG architecture.
- The mathematical framework for specifications of electric engines, factors of solar panels, and energy storage systems (ESS) was evaluated.
- An innovative CHPO method has been utilized to determine the most effective bidding technique for the transaction process and the algorithm performance in various parameters was provided.
- A unique security protocol based on blockchain technology has been implemented to enable secure data transfer and prevent unwanted access during energy transactions, hence improving data integrity and privacy.

### Research gap

- Although there have been tremendous advancements in blockchain technology, its application to optimize MG energy transactions, especially for dynamic and real-time power pricing systems, remains relatively unexplored.
- Existing studies lack insight into collaboration frameworks between MU and UoM in optimizing the efficiency of the transaction process as well as tackling the energy underutilization issues appropriately.
- At present, more studies on scalability and validity for methods like CHPO which were proposed to work well on systems under diverse technologies like microwave, optical, and RF.

The following is the structure of the article: In Stage 2, related works are determined. Stage 3 has the explanation of the proposed method having a mathematical model, computational system, and CHPO method for transaction processes examined. The performance analysis of research participants and the proposed CHPO techniques were demonstrated in Stage 4. Conclusions are explored in stage 5.

## Related works

### Blockchain in energy management

With RE, storage of energy, and flexible loads in virtual power plants (VPP) [11], they provided a blockchain-based platform for VPPs that enables a wide range of transactive power activities among residential users. The blockchain-based VPP energy control applications lower users’ costs and lower system costs overall, as shown by the simulation results. As an invention, blockchain is predicted to have a significant impact on virtually all web-based transactions. Based on the analysis, there has been an increase in integrating blockchain technology with RE [12] throughout the years which suggests that the blockchain studies on energy were focused on RE. The blockchain maturity questionnaire, designed by the research [13] was used to assess the amount of confidence and development of the technology’s implementation. It explored its present level of information on the blockchain, the primary advantages and drawbacks of using it, and the degree to which certain associations were determined to incorporate the technology into their operations. With most research employing qualitative analysis, existing investigations on blockchain + energy are mostly application-focused and fail to reach sufficient distance in examining the state of collaboration between various areas [14]. It utilized modern cartography to perform a methodical source evaluation in the area of blockchain + energy and executed bibliometric analysis with Vos viewer from both quantitative and qualitative perspectives. Research on blockchain and renewable energy focuses on applications and qualitative assessment, but lacks exploration of interplay between blockchain and other energy sectors, limiting understanding. With the use of blockchain and dispersed power resources analyzed through Porter’s five-force model, the feasibility of distributed sources of power was investigated [15]. The strengths, weaknesses, opportunities, and threats (SWOT) model was used to assess the joint development framework of the blockchain + DERs based on the viability analysis, demonstrating the viability of integrating blockchain technology with distributed energy resource systems. The intention of the analysis was to identify areas of unsatisfied research need and potential paths for blockchain-based power distribution [16]. Although there have been developments in blockchain for DERs, research has not studied in depth the obstacles and potential approaches to widespread adoption and improved integration of blockchain technology into power distribution systems. The previous statement highlights the limitations of existing designs and research gaps that require solutions before the technology of blockchain can be extensively employed. The article included an in-depth examination of a DER [17] management and integration applications based on blockchain. An analysis of sources that employ blockchain technology to be concerns related to corporate operations and DER integration. DER incorporation and control issues have been solved among the primary research subjects and have potential with the use of blockchain technology. Firms investigate the methods by which technology and society interact in the energy sector, the blockchain system’s benefits and the processes in power markets, information storage and enhancing transparency in electric power and energy services were examined in research [18]. It explored the potential benefits and drawbacks of blockchain energy. The literature often overlooks the comprehensive evaluation of the practical application of blockchain systems in energy markets, particularly in relation to regulatory contexts and market structures.

### Blockchain in security

To identify the impacts of the photovoltaic model, the research suggested the blockchain-based approach [19]. The creative method includes network detection, network data screening, and confusion assessment in data of in-transit by dispersed records. An asymmetric encryption technique [20] utilized in the experiment. The smart contract secured using both private and public keys employing the experimental data of individuals to obtain the necessary transaction data and enable the associated power distribution to operate during the transaction time. Even though techniques based on blockchain prepare enhance securities by enhancing encryption and through the introduction of smart contracts, there is still a significant need to improve the protections of data to prevent data weaknesses in decentralized systems. The method presented in [21] improved the perception and connection rate of company records significantly by strongly managing the execution of control information and standardizing the management of data usage permissions.

### Renewable energy integration

They employ blockchain technology for wireless networks to create an efficient power market system in the smart grid [22]. The combination of the local power transaction blockchain and the solar power energy trade blockchain in the dual-chain design resulted in increased power investments and RE usage efficiency. The research analyzed these indicators and revealed the complicated relationships by utilizing sophisticated data analytics approaches called the fuzzy Interpretive Structural Modeling (ISM) method and the fuzzy Analytic Network Process (ANP) technique [23]. It provided the establishments intending to take advantage of blockchain innovation and manage the evolving conditions of reliable power chains with a strong foundation. A few researchers evaluating the RE integration using blockchain ignore the intricacies of power transactions, and the complex relationships created by the different variables that impact power investments and the efficiency of RE usage. The research presents an economical model for a predictive control (EMPC) system [24] for the grid-connected MG time-of-use rate application of the powered backup facility and solar system. Blockchain technology was employed to secure data provided in the grid, preventing hackers from affecting data. Through the analysis of big data on electricity usage, power supply techniques can be developed based on the behaviors of electricity users. The two-stage play framework was developed to explain the power demander’s and supplier’s pricing rivalry approach [25]. Blockchain technology is being used in grid-connected MG, but there is limited discussion about scalability, especially when loads deviate from nominal values while expanding geographical areas. The Nash equilibrium result in the initial and final stages of the framework can be obtained by applying the inverse technique, making it quicker to implement the distributed power actions decision-making optimization in the blockchain framework. The system operation of the severe security requirements makes it difficult to reach the standard centralized control of security mode for the severe security that it faces.

This section shows blockchain’s transformative potential in energy management, through applications in VPPs, DER integration, and optimization of RE systems, which benefit from cost saving, transparency, and security improvements. Most studies rely on qualitative analyses and fail to research multi-dimensional collaboration, which is also criticized in bibliometric reviews. Gaps have been identified in regulatory aspects, scalability, and general frameworks for comprehensive integration, which require more robust, data-intensive research to realize the full potential of blockchain in energy systems.

### Blockchain-based energy transactions

By enabling value extraction from excess energy, microgeneration of renewable energy enhances prosumer networks. Tokenized energy assets require a decentralized, access-controlled, and immutable system. There were two versions of the unified blockchain-based system for energy asset transactions that were deployed on Hyperledger Fabric. Tokens that reflect value are modeled as fungible tokens (FT) and assets as non-fungible tokens (NFT). For the majority of significant operations, the system’s performance is comparable, and a thorough comparison of FT and NFT implementations was provided [26]. A permissioned blockchain-based energy management mode for microgrids powered by renewable energy is proposed. To prevent ineligible participants, the system employs entity mapping with distinct identities for every business, individual, or gadget. Although each peer entity only retains its own unique transaction information, everyone helps maintain the network’s transaction information index. This solution can prevent communication lags and encourage plug-and-play because the permissioned blockchain is distributed. A Hyperledger Fabric permissioned blockchain demonstration program is used to assess the effectiveness of the suggested approach. According to simulation results, the suggested approach is feasible and supports P2P energy management and privacy protection for decentralized energy systems [27].

### Research gap

The previous studies have some limitations like limited scalability of blockchain fields for large amounts of energy transaction management. More insufficient energy and expensive to function that related with blockchain mechanism. Complex in evaluating the long-term impacts of blockchain technology in energy systems and difficulties with assessing the appropriate data for market performance. Developing energy landscapes is complicated to accurately predict the competitive dimension. There is a limitation in the lack of providing external components that affect the energy system. Blockchain reliability was affected by complications in wireless network security. Lack of maintaining the blockchain networks as parallel and had technical interoperability between various DER management systems. Reduced numerical validation of fuzzy analytical network process outputs in energy framework. There are more security issues in IoT device transmission. Uncertainties in economic feasibility estimation for blockchain based MG systems. Reduced transaction speed in the power market and maintaining regulatory compliances is complex at the time of enhancing the data security measures. The research collectively demonstrates the integration of blockchain technology with RE and power systems, determines the advantages including efficient enhancement and cost reduction. It has issues with security and standards and lacks extensive cross-disciplinary collaboration. The emphasis continues on applications, with minimal consideration provided to exploring wider implications and the relationship between technology and social factors. Research suggests the utilization of blockchain technology in combination with MG to solve these issues of energy waste in a MG competing competition. This research fills these gaps by creating a blockchain-based architecture that integrates with MG to improve scalability and transaction efficiency in energy systems. By applying innovative optimization approaches, research increase blockchain-based MG systems’ data security, interoperability, and economic viability. Furthermore, the suggested model promotes cross-disciplinary cooperation, allowing for a comprehensive approach to energy management and social implications. Despite encouraging outcomes, there are remaining inquiries about the long-term performance of tokenized energy trading, interaction with current grid infrastructure, and real-world scalability. Furthermore, more research is needed to guarantee the successful implementation of such decentralized energy management systems in a variety of regulatory contexts, as well as compatibility across various blockchain platforms, dynamic pricing mechanisms, and user adoption. By facilitating robust optimization in decentralized systems, dynamic flexibility in prices, and speedier convergence, the suggested CHPO approach addresses these difficulties. Energy transactions are guaranteed to be safe, scalable, and private due to its blockchain integration. CHPO is perfect for managing tokenized assets in a variety of regulatory-sensitive micro grid scenarios because of its flexibility, which facilitates interoperability and real-time decision-making.

### Mathematical modeling & validation of photovoltaic (PV) model

PV forecasting yields either photovoltaic power or solar irradiance. Predicting PV power and energy management requires knowledge of solar power generation characteristics such as system factors and forecast horizon. The creation of new forecasting models and predictors for solar energy is aided by standardized performance evaluation measures.

### PV generation

The measurement of solar irradiance, reflectance, estimation of PV cell temperatures, and other parameters all have an impact on the expected PV power production. Modeling the highest power output is done by Equation (1):


$$P_{V}=\eta AI[1-0.05(t-25rbrack$$ (1)

Where A is the array area $\left(m^{2}\right)$, I is the solar irradiance ($kW/m^{2}$), t is the outside air temperature (∘C), and η is the PV array’s conversion efficiency (%).The simulink model parameters are as follows: open-circuit voltage $V_{oc}\;=\;21.6\;V$, shunt resistance $R_{sh}\;=\;1000O$, series resistance$R_{s}\;=\;0.0001O$, number of series cells$N_{s}=\;36$, number of parallel cells $N_{p}\;=\;1$, maximum power $P_{\max}\;=\;105\;W$, voltage at maximum power $V_{\max}\;=\;18.46\;V$, current at maximum power Imax = 5.74A, short circuit current $I_{sc}\;=\;6.11A$, and series resistance $R_{s}=\;0.0001O$.

### Maximum power point tracking (MPPT)

The implementation of this method involves the addition of a PI controller, which can enhance the performance of the incremental conductance (IC) MPPT by decreasing the error between the incremental conductance and the actual conductance. This allows the necessary adjustment to be made and the system to be updated gradually as needed. The perturb and observe (P&O) MPPT technique’s problems, such as its oscillation around the MPP under rapidly changing atmospheric conditions, are also addressed by this PI controller. The increment conductance of the PV module determines whether or not MPPT has achieved the MPP. It also establishes stopping conditions for perturbation at the operational point. The relationship between dI/dV and I/V could be used to determine the MPP in this MPPT approach. When the maximum power point is on the right side of the P-V curve, the slope dP/dV is negative; similarly, when the MPP is on the right side, it is negative. This MPPT’s benefit is that it takes less time to reach the MPP and is more effective at handling disruptions and changes in the environment. The ability to determine which way to adjust the PV generator’s operating point to get it closer to the MPP is the main advantage of the IC MPPT over the P&O MPPT. It will not deviate from course in rapidly changing weather conditions because of this, and the working point does not fluctuate about the MPP after it has been achieved.

### Methodology

This research aims to create and apply the Customized Hunter Prey Optimization (CHPO) algorithm in a blockchain-based MG transactional framework for optimal energy transactions and improved market participants’ performance. The approach solves the challenges of energy under the use and real-time power pricing in the MG systems. This research ensures secure, transparent, and efficient transactions between MU and UoM through the application of Blockchain Technology. The system is also being investigated for applicability across various technologies which improve scalability. It allows the seamless integration of DERs in MG environments. This research seeks to enhance energy transactions using CHPO algorithm with blockchain technology in MG By employing the CHPO algorithm, efficient and effective approaches can be identified for the MU and UoM to optimally buy and sell power in real-time. This resolve improves the performance of the market overall. Blockchain integration enhances security, transparency, and real-time power pricing in DERs, promoting RE integration in MG and enhancing economic outcomes through reliable infrastructure.

### Data collection

This research gathers the Blockchain-Enabled Microgrid Transactions Dataset to optimize the bidding tactics in a decentralized market. The data has been gathered from the Kaggle platform (https://www.kaggle.com/datasets/ziya07/blockchain-enabled-microgrid-transactions-dataset/data). It is a tool for research the trading of energy, identity verification, and data protection in solar power generation regulation.

### Biding approach using transaction process through customized hunter prey optimization (CHPO)

In solar power generating data security, the bidding approach is to increase the encryption and verification process, enabling secure data transfer and avoiding unnecessary access to operational data. Utilizing the low and high velocities proportions technique, an algorithm has been developed. To solve this issue of optimum values getting constrained in local minima is the target of this modification. There are two phases to the approach: the extreme velocity ratio and the minimal velocity ratio. The initial phase is represented by an enormous advance size, suggesting an increased level of exploratory ability. It can be mathematically expressed as Equations (2–4).


$$iter<\frac{1}{3}Maxiter$$ (2)

$$T={\vec{Q}}_{Y}⨂(F-{\vec{Q}}_{Y}⨂A_{j}\left(s)\right)$$ (3)

$$A_{j}(s+1)=A_{j}(s)+O.{\vec{Q}}_{Y}⊗T$$ (4)

The vector${\vec{\;Q}}_{Y}$, indicating Brownian motion, is comprised of random integers selected from the normal distribution. Multiplication between elements is represented by the symbol ⊗. A constant O= 0.5 and a vector of consistently random numbers that range from 0 to 1 constitute ${\vec{Q}}_{Y}$ that is multiplied by the previous location to create the new location. The significant amount of exploration capacity is indicated by the step size being huge in this instance which occurs in one-third of the rounds. The initial T stands for the maximum iteration and the sign s for the present iteration. J is the directory of the mediator or resolution in the population. S is the iteration phase or total in the optimization procedure. The optimal location is determined by utilizing the fittest solution, represented by (F) to create a matrix. The optimal solution, denoted by Ay in the equation is repeated m times to produce the F matrix. m and c represent the number of search engines and dimensions as represented in Equation (5).


$$\begin{array}{c} F=\left[\begin{array}{c} {{Ay}^{s}}_{1.1}\cdots {{Ay}^{s}}_{1.c}\\ \;\vdots \;\ddots \;\vdots \\ {{Ay}^{s}}_{m\mathrm{.1}}\cdots {{Ay}^{s}}_{m.c} \end{array}\right] \end{array}$$ (5)

The second phase of optimization known as the low-velocity ratio occurs toward its end and is frequently linked with greater exploitation capability. The Lévy flight is considered to be the most effective technique. The following Equations (6–8) are the explanations for this stage:


$$iter>\frac{1}{3}Maxiter$$ (6)

$$T={\vec{Q}}_{K}⨂({\vec{Q}}_{K}⨂F-A_{j}(s))$$ (7)

$$A_{j}(s+1)F+O.DE⨂T$$ (8)

**Fig 2:**
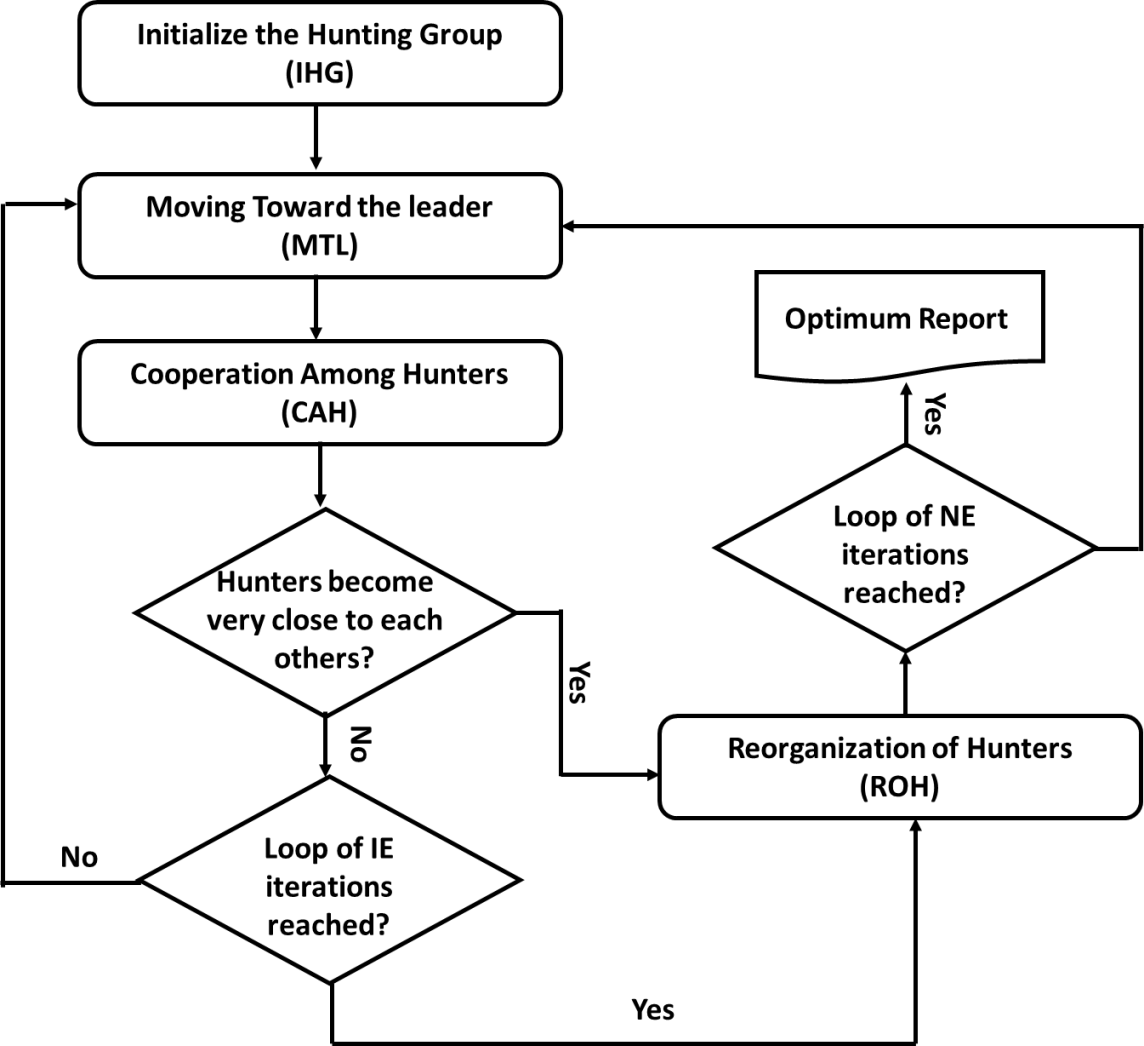
Flow chart of CHPO.

By multiplying ${\vec{Q}}_{K}$ and F and then inserting the step size, the Lévy technique updates the location. Increasing the possibility of preventing local optima is one of CHPO’s extra advantages. Fig 2 depicts the flowchart for the CHPO. The CHPO’s ability for exploration has been improved by this modification as shown in algorithm 1.

*Algorithm 1: CHPO*

*Initialize the process*

*Initialize the variables in the optimization*

*Develop the primary blockchain installation*

*Estimate the blockchain installation*

*While (*j<Maxiter)

*Estimate the level of provided blockchain design*

*Modify the variables of blockchain security*

*Estimate the security design*

*If (security criteria are not reached)*

*Modify the protocols of security*

*Estimate the security of access and the reliability of data*

*Modify the security measures and blockchain*

*Return improved security installation*

*End*

### Computational system for market participants

Blockchain is quickly emerging in energy transactions to the power sector, as indicated by its usage in the financial sector for digital currency. Considering the majority of the power grid’s electricity obtained and selling is distributed. With the use of blockchain technology and a significant number of MG participants, transactions are concluded directly between sellers and buyers, improving the position and market impact of the previous employer. MG trading involves the distribution of transactions containing bidding information by operators and users into the trading area. The system creates smart technology by utilizing the information provided by both sides. Research is selected two market participants for the performance such as Users of Microgrids (UoM) and major users (MU). The market performances are creating various methods for buying and selling power at various locations in the MG framework to maximize their own interests and influence developments in the market. Since the goals and demands of the three are diverse, each needs a specific functional connection for development. The security and dependability of transactions can be enhanced by the characteristics of data transparency, tracking, and prevents attackers from altering the code of software applications. Blockchain technology combines transactions based on the amount of power and price. It enables the relationship between nodes and encrypts the transferred data for dependability and security. The technology enhances the overall effectiveness of MG energy management by providing high efficiency, accurate data traceability and timely allocation of electricity.

**Users of Microgrids (UoM)**: The MG output processes vary depending on the demand, it utilizes intermittent energy sources (solar, wind, etc.) in a particular period of time, and external radio frequency power is excess or insufficient. To generate earnings$O_{sell}^{UM}(s)$, the UoM can sell excess power to other consumers. The following is an Equation (9) for$O_{sell}^{UM}(s)$.


$$O_{sell}^{UM}(s)=\sum_{j=1}^{m_{1}}o_{sell}^{NtoN_{j}}(s).r_{sell}^{NtoN_{j}}(s)+\sum_{i=1}^{m_{2}}o_{sell}^{NtoC_{i}}(s).r_{sell}^{NtoC_{i}}(s)+\sum_{n=1}^{m_{3}}o_{sell}^{NtoK_{n}}(s).r_{sell}^{NtoK_{n}}\;(s)$$ (9)

The radio frequency of electricity selling price to the $j^{th}$UoM and $n^{th}$MU at time s is represented by $o_{sell}^{NtoN_{j}}(s)$ and $o_{sell}^{NtoK_{n}}(s)$. The amount of power sold to the $j^{th}$ UoM and the $n^{th}$MU at time s is represented by $r_{sell}^{NtoN_{j}}\;(s)$ and $r_{sell}^{NtoK_{n}}\;(s)$ each.

**Major Users (MU)**: Large consumers require a minimum amount as possible to provide the desired amount of power. It makes an inference that the systems of multi-Microgrids primary consumers have the necessary resources to generate power to simplify it. The main objective for major users searching for power is to determine the cost of performing through UoMs$D_{buy}^{NtoV}(s)$. The particular method of purchasing power is determined by each entity’s procedure for purchasing electricity and the mathematical Equation (10) is as follows.


$$D_{buy}^{NtoV}(s)=\sum_{j=1}^{m}d_{buy}^{N_{j}toV}(s).r_{buy}^{N_{j}toV}(s)$$ (10)

Within these, the price unit and energy quantity of purchasing on the $j^{th}$ UoM at an instance are represented by $d_{buy}^{N_{j}toV}\;(s)$ and $r_{buy}^{N_{j}toV}(s)$.

### Microgrid-Based Renewable Energy Trading System

Qualified power producing businesses, power sales companies or large consumers can ascertain transaction capabilities and transaction costs through bilateral or multilateral transactions, consideration the inherent relationship between dispatching operations and market transactions. By utilizing MG as a virtual power plant, numerous sources within the distributed power-delivering system can be connected to establish a complete participating power market. User appliances, energy storage systems, control systems, and distributed power generating systems based on RE could be independently combined by the MG. Table 1 displays solar panel factors. The efficiency of the solar panels and the incident sun irradiance limit the power output I of solar panel $P_{t}$at time t in solar power systems. The power limitations of $P_{Mimax}(t)$and $P_{Mi\;min}(t)$ are contingent upon the amount of solar radiation received and change with time. They are represented as $P_{Mi\;min}(t)$ and $P_{Mimax}(t)$ (Equation 11). The solar panel’s characteristics and external factors that affect solar energy conversion, including clouds and shade, determine the limitations.

**Table 1:** Factors of Solar Panel.

	Solar Panels	Power
Solar Panel Identifier	Number 1	30
	Number 2	25
	Number 3	20
	Number 4	20
Power Unit		Watts (W)


$$P_{Mi\;min}(t)\leq P_{Mi}(t)\leq P_{Mimax}(t)$$ (11)

Solar panels are included as the users of the approach and electric engines are utilized by major consumers with power not sold, since the deployment of innovative environmental protection technologies that include quick charging power storage and electric engines in the MG Table 2 depicts the factors of electric engines and ESS. $S_{oc}$Represents the electric car battery’s state of charge at the completion of the $t^{th}$ phase, η denotes the battery’s efficiency in both charging and discharging, $E_{r}$ denotes its rated capacity, and $S_{ocmin}(t)$ and $S_{ocmax}(t)$ denote the maximum and minimum levels of charge. Equation (12) determines an electric engine.

**Table 2:** Factors of Electric Engines and ESS.

Factors		Values
Specifications of Electric Engines	Effectiveness of Charging	90%
	Effectiveness of Discharging	95%
	State of Charge ($S_{oc}$) initialization	0.5
	Maximum $S_{oc}$ ($S_{ocmax}$)	0.95
	Minimum $S_{oc}$ ($S_{ocmin}$)	0.4
	Potential	25
Factor of ESS	Voltage (V)	626
	Speed in Charging and Discharging	95%
	Current (A)	200
	Potential	100


$$\left\{ \begin{array}{c} S_{oc}(t)=S_{oc}(t-1)-\frac{t}{E_{r}}\eta \\ S_{ocmin}(t)\leq S_{oc}(t)\leq S_{ocmax}(t) \end{array}\right.\;$$ (12)

Variation in charging loads can be minimized by providing utilization of ESS at fast-charging stations. Q(t) represents the power storage level of the system at time t. Three electric vehicles, one electric energy storage device, and four solar panels constitute the MG. The maximum and lower bounds of the power storage engine are denoted by $Q_{\min}(t){\;and\;Q}_{\max}(t)$ in Equation (13).


$$Q_{\min}(t)\leq Q(t)\leq Q_{\max}(t)$$ (13)

In general, the capacity of the photovoltaic and Energy Storage System (ESS) can vary greatly depending on the microgrid system and its design purposes. PV Capacity indicates 5 kW to 1000 Kw and ESS Capacity represents 10 kWh to 5000 kWh, depending on the size of the energy storage system. The depth of discharge (DOD) of lithium-ion batteries is usually in the range of 80% to 90%. This means that only 80–90% of the total energy capacity of the battery can be used before recharge is required.

## Performance analysis

### Experimental setup

The proposed configuration has been evaluated using Tensor Flow version 1.15.0 in Python with a Core i7 CPU and 24 GB of RAM as machine requirements.

### Evaluation criteria

#### Outcome of suggested method.

The objective function is optimized and the effectiveness of the CHPO in the MG model transaction is tested in the experiment by evaluating the proposed technique. Establish the function’s output as the difference between the highest UoM and the lowest UoM values. The research uses Metrics such as mean time of convergence, Iteration interval, Scalability, Latency, Lowest UoM and Highest UoM to support the reliability of the results. Table 3 presents the outcome of the proposed CHPO.

**Table 3:** Outcomes of the Proposed CHPO.

Metrics	CHPO (Proposed)
The mean time of convergence	54
Iteration interval	240
Scalability	Highest
Latency	45ms
Lowest UoM	2402077
Highest UoM	293589

**Fig 3:**
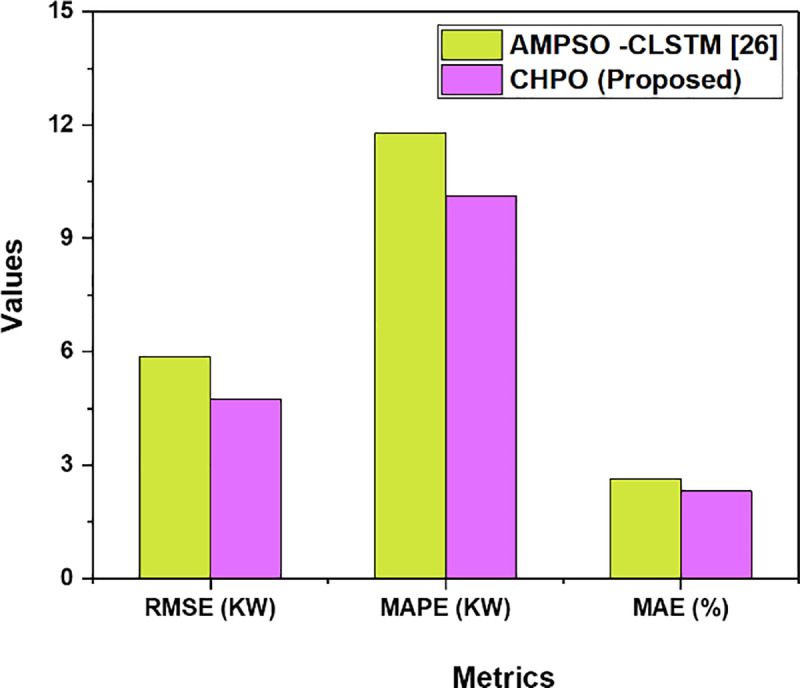
Final Outcomes.

The uses of metrics such as RMSE, MAP, MSE for comparing the Proposed CHPO with the existing method such as adaptive mutation particle swarm optimization-convolutional long short-term memory network (AMPSO -CLSTM) [28] as show in Table 4 and Fig 3. RMSE calculates the square root of average squared differences between the actual and predicted values. In the context of a blockchain-based photovoltaic system, RMSE could help evaluate the accuracy of forecasts in energy consumption or prices. MAPE calculates the average absolute percentage errors between the predicted and actual values, thus providing an intuitive measure of forecasting performance. It is very useful in measuring real-time energy pricing models within blockchain systems, where the percentage error is important in decision-making. MAE computes the average absolute difference between the predicted and observed values, giving equal weight to all errors without any amplification of outliers. In identity authentication and blockchain-based transaction models, MAE can evaluate the quality and reliability of data and predictions.

**Table 4:** Final outcomes of Proposed CHPO.

Metrics	AMPSO-CLSTM [28]	CHPO (Proposed)
RMSE (Kw)	5.87	4.75
MAPE (Kw)	11.78	10.12
MAE (%)	2.64	2.31

#### CHPO in blockchain based microgrid optimization.

**Fig 4:**
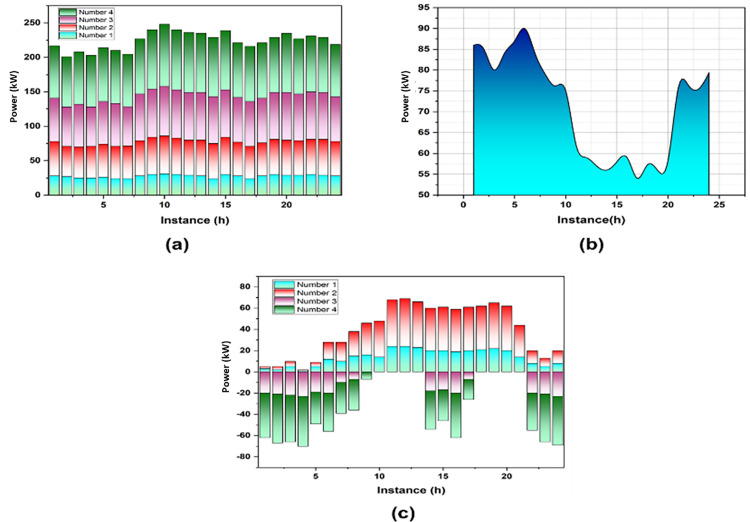
Outcomes of(a) solar panel, (b) ESS and (c) electric engines.

On the blockchain, each node has an identical state, and a system of coordination functions together to keep processes functioning effectively. Utilizing a novel CHPO method contributes to the variables in the solar panels, a system of storage for energy, and electric engines through experimental simulation. The output power of each topic in the simulation of small MG is output and outcomes are displayed in Fig (4a-c).

#### Market dynamics in power exchange: UoM and MU interactions.

**Fig 5:**
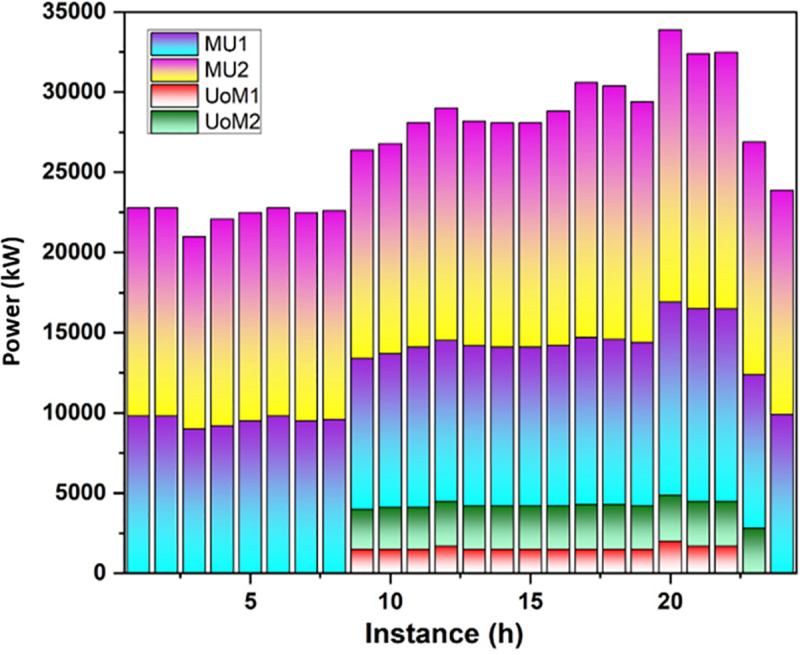
Electricity demand on the entity ofthe market.

**Fig 6:**
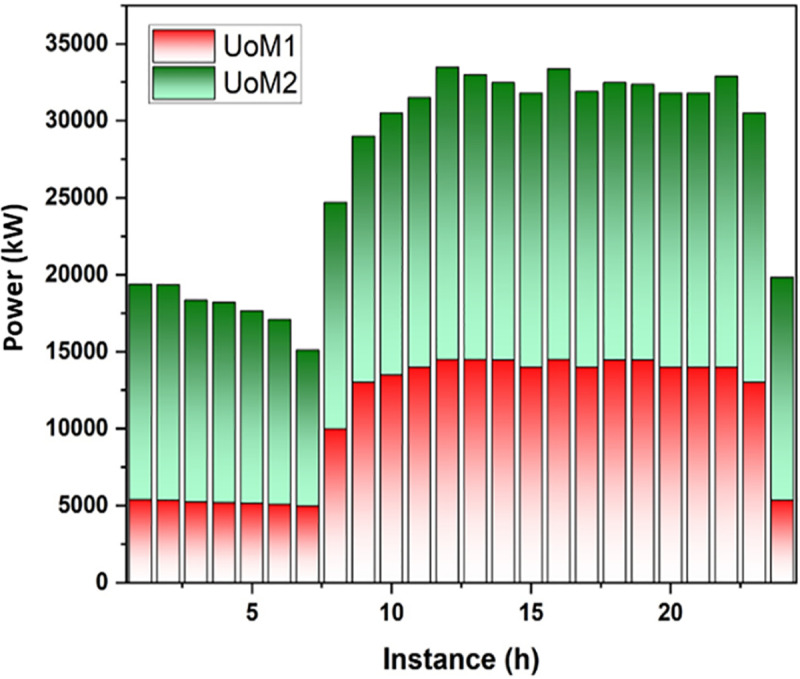
Power Supply of UoM in one day.

**Fig 7:**
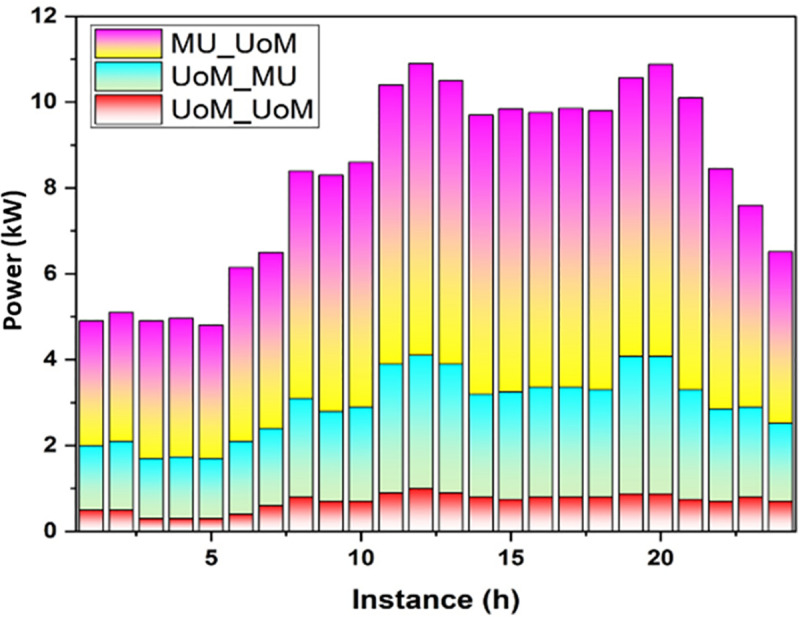
Highest earnings ofUoM.

Two categories of MUs and two categories of UoMs have been selected by the system as the primary participants in the market activity. The activities determine that the power purchase amount for each participant determined gets replaced with the corresponding power selling price and they interact with one another based on their specific desires. Set quotation methods at random for each market entity among bidding technique limits and provide the optimum component and maintain the constant. In the framework, the highest number of repetitions and the overall number of entities in the market (N = 4) are set. This research uses UoMs to confirm the model’s viability as it is a multiple objective, multiple stage technique issue. Analyzing the data reveals each group’s one-day power demand and the amount of electricity frequency provided by UoMs. Figs 5 and 6 depict each market group’s one-day supply and demand for power:

Fig 7 illustrates that there is a strong demand for power throughout the day and all market participants charge more for it. There is a small difference between the estimates of UoMs and MUs when it comes to competitiveness. UoMs are MUs’ main power suppliers as a result and MUs have received greater benefits and larger estimates from UoMs. Table 5 shows that the point of the system demand impacts its maximum and a few UoMs without power can purchase radio frequency from other sources.

**Table 5:** Transactional Quote Instance.

Transaction Participants	Power price optimum ($)
UoM_MU	1.04
MU_UoM	1.05
UoM_UoM	1.06

**Fig 8:**
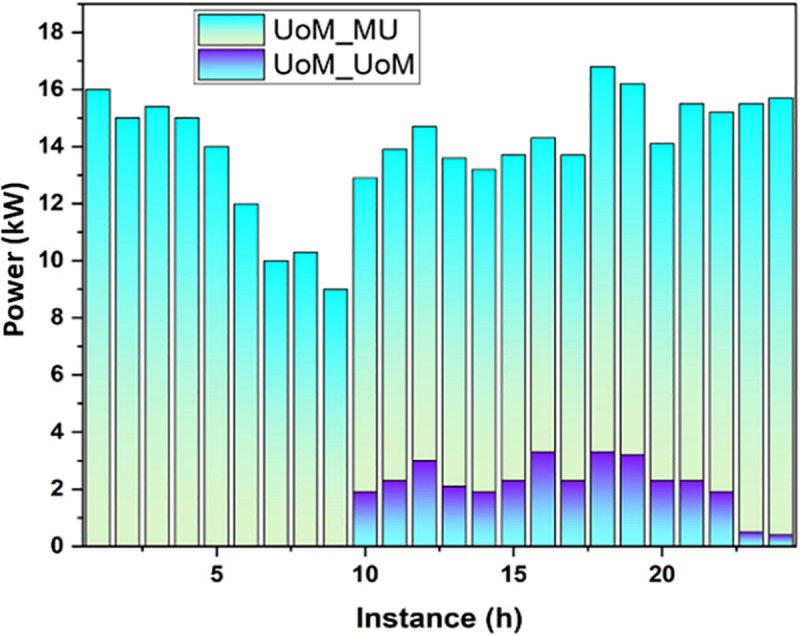
Trading Electricity.

Fig 8 shows that the low usage of radio frequency level is experienced by each market entity during one day. Each power provider refuses to provide power to UoMs, and there is minimal demand from major companies.

### Comparative analysis

A comparison of key performance metrics is made between the suggested CHPO technique and the current algorithm PoW-GAD [29].

### Optimization algorithms performance in computation time

**Fig 9:**
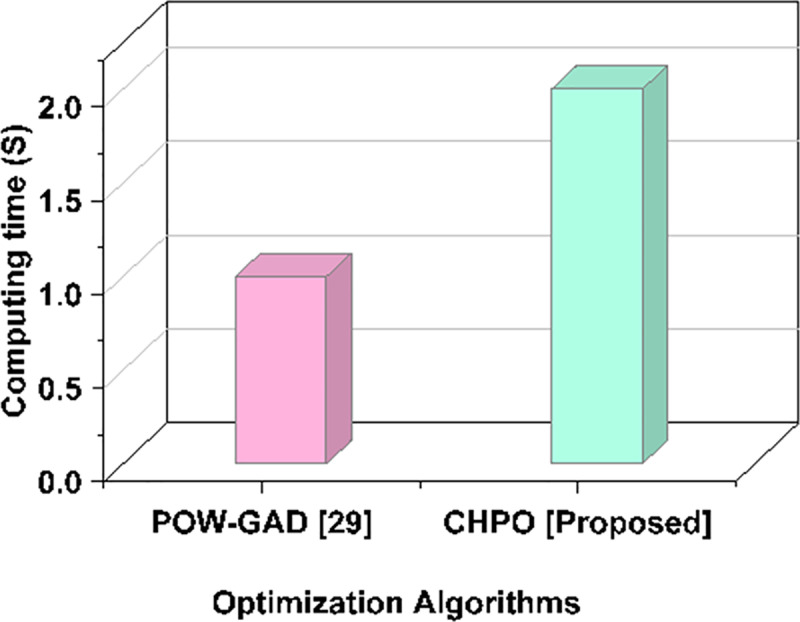
Comparative Outcomes ofComputation time.

Significant variations can be observed when comparing the computation durations for various encryption and authentication techniques. While offering improved security through decentralized verification, blockchain-based solutions typically take longer to compute than conventional centralized systems. This trade-off is, however, justified by the increased resilience to cyberattacks, which guarantees the integrity and safe access to vital data in solar power control systems. Table 6 and Fig. 9 depicts the comparison outcomes of computation time (s).

**Table 6:** Comparative Outcomes of Computation time.

Optimization Algorithms	Computation Time (s)
PoW-GAD [29]	27.19 s
CHPO [Proposed]	20.15 s

When comparing optimization algorithms for identity identification in solar power production, the suggested CHPO approach drastically cuts the calculation time to 20.15 seconds, whereas the PoW-GAD [29] strategy takes 27.19 seconds. This illustrates how the CHPO has improved efficiency, providing a quicker option while preserving the efficacy of security in blockchain-based applications.

## Discussion

The research demonstrates the market dimensions between two main market participants that include MU and UoM in power generating methods. Both categories were determined through transaction management through the desire of bidding by the participants. By utilizing the MU technique, the connections of the supply, indicating the highest earning of power and demands for powers are examined. The MU provides the competitive earnings and advantages of UoM and significant participants in MU. Transaction assessment explored the instance of power cost. The prices collected are descriptive of the level of competitiveness between market participants. As it involves efficiency, there is a moderate difference between the UoM and MU estimations. MUs have benefited more and acquired larger estimates from UoMs that serve as the primary power suppliers. Each market participant gets the low radio frequency level utilization in one day. There is modest demand from major firms and all power suppliers decrease to deliver power to UoMs. Each node on the blockchain exists in the same state and an integration system functions in connection to maintain the efficient operation of activities. The CHPO approach has major implications for real-world applications, as it can improve energy management in microgrids, optimize bidding techniques, and increase power transaction efficiency. This technique has the potential to transform energy trading by allowing for real-time price modifications and minimizing transaction costs. The effective implementation of this strategy can also aid in the integration of RE sources, boosting sustainability while preserving economic competitiveness. Significant up-front setup costs and legal barriers to data and energy market regulation can hinder the deployment of blockchain in photovoltaic management. This can only be overcome through stakeholder collaboration and proper regulatory design. The blockchain framework using CHPO offers superior efficiency compared to other existing methods with rapid convergence, lower latency, and improved scalability to large microgrid systems. Its decentralized nature also contributes to enhanced security because there are no single points of failure, providing better resilience against cyber threats. By using an innovative CHPO technique, experimental simulation is utilized to improve the factors in solar panels, energy storage systems, and electric engines. The experiment assesses the effectiveness of the proposed method in the microgrid system transaction. The AMPSO-CLSTM method [28] has limitations, including slow convergence of AMPSO, potential local optima issues, and the complexity of the combination of PSO and CLSTM. This leads to longer computation times and inefficiencies, especially in real-time applications. Additionally, the combination of PSO and CLSTM makes training the model difficult and requires significant computing power. The CHPO algorithm, combining blockchain technology, offers several advantages over AMPSO. It converges faster, allowing for more efficient optimization, especially in MG systems. CHPO avoids getting fixed at an optimum, improving performance and optimization. Blockchain technology also enhances the dependability and integrity of energy transactions, making it more scalable, efficient, and secure for real-time power pricing and management in distributed energy systems.

The blockchain-based CHPO model significantly enhance microgrid energy transactions. Since energy laws differ from one area to another, there might be regulatory challenges with the model. While the model is cost-efficient, high setup costs would limit its acceptance for smaller players. As for scalability, the model seems capable, however, further testing and adaptation of the model with diverse, large-scale microgrids would be required through real-world scenarios. The future implication of the proposed model aims to enhance energy transaction efficiency and transparency in decentralized grids, potentially promoting blockchain use in renewable energy markets for real-time power pricing. It also suggests scalable integration of distributed energy resources for grid management.

There are a number of obstacles in the way of the CHPO model’s actual application in energy markets. Legal frameworks differ by area, raising regulatory issues that could limit blockchain-based energy trading. Economic viability is another issue because the expensive setup and maintenance costs can discourage startups. Moreover, it grows increasingly complicated to guarantee compatibility with current grid systems. The management of large-scale micro grids with a variety of energy sources and consumption patterns is a challenge to scalability. For deployment to be effective, real-time data processing, secure transaction handling, and stakeholder participation are necessary. Standardized rules and financial incentives can be essential for removing these obstacles and promoting wider use.

The research addresses energy underutilization and real-time pricing, two issues that are frequently disregarded in current frameworks, by introducing a revolutionary CHPO algorithm that is integrated with blockchain. It focuses more emphasis on cooperative negotiating between major users and microgrid users than existing models, which allows for improved scalability, security, and dynamic flexibility.

## Conclusion

To discover the most effective bidding method for the transaction process, the research examined the issue of individual earnings. A local multiple microgrid market participants’ model based on blockchain technology was created by examining the needs of various market participants, including UoM operators and MU. The CHPO was utilized to perform an appropriate examination of blockchain transactions based on the competitive interaction between each participant and their separate desires. Improved identification and convergence capabilities of the customized algorithm enhance the efficiency of the outcome. Internal power transactions in the microgrid can be performed by the blockchain-supported transaction system that has been offered in the research. There is an indication value for choosing electric vehicles and solar power generation based on the real model. The microgrid can have more dispatchable resources and clean energy with the large growth in the percentage of load flexibility and the advancement of demand management technology. The participants of the transaction are very flexible and efficient, and they can both dynamically modify the valuation in response to market transaction information. CHPO provides higher iterative and search ability capabilities and can be utilized more effectively for multi-objective solution models by increasing the development factor.

### 5.1 Limitations and future scope

The limitations of the proposed approach require the major processing power and need more possible scalability. Future research could utilize the developed method to optimize more power systems, including integrated power and heat dispatching, optimal power flow and examine useful applications in various energy markets to improve system stability and flexibility. It could be expanded to optimize the location of solar power sources in transmission and distribution networks, considering its efficacy in determining the optimal electrical features of photovoltaic energy. The CHPO model, which is integrated into the blockchain, can scale by its use of private blockchains for local transactions and public ones for cross-grid trading; it is interoperable owing to support for various energy sources with standard protocols. Economically, optimum bidding strategies are used to reduce transaction costs. A roadmap includes widening from single Microgrids to regional grids, enhancing both system stability and flexibility.
